# Assessment of the Exposure to Gradient Magnetic Fields Generated by MRI Tomographs: Measurement Method, Verification of Limits and Clearance Areas through a Web-Based Platform

**DOI:** 10.3390/ijerph18073475

**Published:** 2021-03-27

**Authors:** Riccardo Di Liberto, Daniele Andreuccetti, Moreno Comelli, Giancarlo Burriesci

**Affiliations:** 1 IRCCS, Scientific Hospitalization and Care Institute, Policlinico San Matteo, Viale Camillo Golgi 19, 27100 Pavia, Italy; R.DiLiberto@smatteo.pv.it; 2 IFAC-CNR, Nello Carrara, Institute for Applied Physics of the Italian National Research Council, Via Madonna del Piano 10, Sesto Fiorentino, 50019 Florence, Italy; D.Andreuccetti@ifac.cnr.it (D.A.); comelli@ifac.cnr.it (M.C.); 3 Italian Workers’ Compensation Authority (INAIL), Physical Agents Laboratory, Department of Occu-Pational and Environmental, Medicine, Epidemiology and Hygiene, Via di Fontana Candida 1, Monte Porzio Catone, 00078 Rome, Italy

**Keywords:** MRI gradient magnetic field measurement, clearance zone, exposure levels, risk assessment

## Abstract

This work is the result of a campaign of measures of exposure levels to magnetic field gradients (GMF) generated by magnetic resonance imaging (MRI) tomographs, to which both healthcare staff and any persons accompanying patients who remain inside the magnet room are exposed while performing a diagnostic Investigation. The study was conducted on three MRI tomographs with a static magnetic induction field up to 1.5 T installed in two hospitals of Lombardy. The study aims to characterize electromagnetic emissions within the magnet room and the definition of a measurement method suitable for assessing the level of exposure of healthcare personnel and any persons accompanying patients. The measurements performed concerned the determination of the weighted peak index for magnetic induction, due to the diagnostic GMF, relating to the action levels for the workers and the reference levels for the general population, in force in the European Union. Thanks to the defined experimental setup, the use of two different measuring instruments, and the software resources of the WEBNIR platform, it was possible to identify, for both categories of exposed persons, the “clearance” space, i.e., the distance from the magnet of the tomograph that guarantees health protection concerning the exposure to GMF, according to the indications of the standards in force. The method used showed that the exposure levels to GMF are substantially safe for professionally exposed workers who do not carry specific risks. For workers particularly sensitive to the specific risk, as well as to individuals part of the population, it is however advisable to maintain a distance from the magnet of about one meter to prevent sensorial neuromuscular stimulation effects.

## 1. Introduction

This work describes the measurement and data processing methods as well as the results of a campaign of measurements carried out in two public hospitals in Lombardy. The survey covered exposure to gradient magnetic fields (GMF) produced by the operation of three different magnetic resonance imaging (MRI) tomographs with superconducting magnets of different geometry and static magnetic fields up to 1.5 T. As is known, the GMF are time-variable fields generated by special “gradient” coils inside the tomograph [1]; they have the purpose of locally modifying the main static magnetic field (and therefore the resonant frequency of the atomic nuclei), to spatially encode the signal with which the diagnostic image is generated.

It should be borne in mind that the investigation of minors or problematic patients through with MRI involves carrying out the diagnostic examination under general anesthesia or sedating the patient, therefore in the presence of health personnel who monitor the patient’s health status as well as any accompanying persons.

The main purpose of this work was to identify for the two categories of exposed people, health personnel (workers) and patient carers (population), the distance from the tomograph magnet that guarantees health protection for exposure to GMF, according to the indications of the current legislation.

The objectives of the survey were the following:

1. Investigating exposure levels of population and workers to GMF, in some typical conditions and operating positions—in particular, with a view to applying the European standards and guidelines. MRI tomographs represent one of the most relevant sources of electromagnetic fields in healthcare environments. Although such measurements were already performed in the past [2,3,4,5,6,7,8], the new data acquired makes it possible to increase knowledge of this type of exposure.
2. Comparing, from a radiation protection standpoint, both apparatuses are structurally very different and the results provided by different instrumental chains.
3. Testing the processing and calculation tools made available by the platform WEBNIR, developed as part of a research project [9].
4. Validating the indications (where available) provided by the manufacturers of the tomographs in their technical documentation concerning exposure to electromagnetic fields.

The exposure limits referred to in this work are the action levels (ALs) and the exposure limit values (ELVs) of the European Directive 2013/35/EU, Table 1, for occupational exposures [10] and the reference levels (RLs) of the European Recommendation 1999/519/EC, Table 2, for the general population [11]. Both documents have been adopted by the European institutions following the ICNIRP guidelines [12], which consider the stimulation effects related to the central and peripheral nervous system.

These limits arise from experimental studies that also take into account the response of the central and peripheral nervous system and are adopted in Europe to guarantee, with a precautionary approach, the overexposure of workers and the population.

An in-depth study of the subject is beyond the scope of this study, which has limited itself to adopting them.

Long-term effects of EMF exposure are not currently considered to be established, and the proposed guidelines explicitly protect against short-term effects only. However, compliance with these guidelines will certainly give greater protection also to individuals exposed over time.

## 2. Materials and Methods

### 2.1. MRI Equipment Examined

The reported survey involved three MRI tomographs with two different types of patient exposure. The first with a 1 T superconductive open magnet, the second is comprising two closed cylindrical magnets with 1.5 T superconductive devices. The three tomographs are used for routine diagnostic tasks: the first (A) is a 1 T Philips Panorama tomograph, operational since 2007. The second (B) is a 1.5 T Philips Ingenia tomograph, operational since 2017. The third (C) is a 1.5 T Siemens Magnetom Aera tomograph, operational since 2018.

### 2.2. Measurement Method

**Figure 1:**
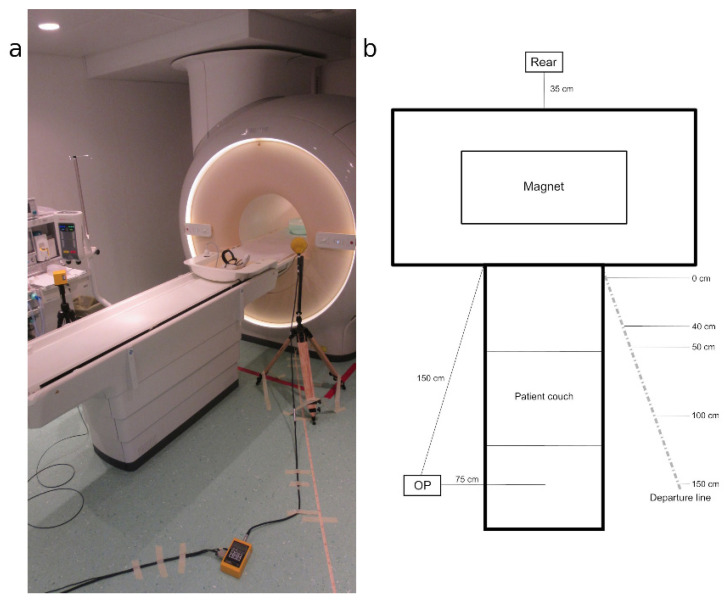
Position of the measuring points: the “OP” and “REAR” points and the measuring points along: (**a**) Real setup; (**b**) Explanatory scheme.

The measurements concerned the magnetic induction dispersed by the gradient coils of the three tomographs; measurements of the electric field emitted by the same coils were also performed, but they provided less significant results with respect to the magnetic field and, for the sake of brevity, will not be reported in this work. The measurement procedure took into consideration a “departure line” from the front mouth of the gantry (in which the patient is inserted) of each tomograph towards the entrance door to the magnet room, with an inclination of about 35° with respect to the longitudinal axis of the patient’s bed (Figure 1).

The measuring points where the probes were positioned were chosen at 100 and 160 cm from the floor and, along the departure line, at 0, 40 or 50, 100 and 150 cm from the point closest to the gantry. The measurement point identified with “OP” is located on the opposite side of the patient’s bed, and the “REAR” point is at the rear of the gantry, aligned with the axis of the bed itself, which is 35 cm from the edge of the back of the magnet.

For each tomograph, some preliminary tests were carried out to identify, among the diagnostic sequences used in the clinical routine, those associated with the highest levels of exposure to GMF. These were then maintained for each measurement session, in order to ensure their reproducibility and comparability (Table 3).

### 2.3. Clearance Areas Around the Gantry

Although it is not common practice to remain inside the magnet room during diagnostic acquisitions, as already mentioned, the presence of healthcare personnel and/or carers may occasionally be required for special needs (for example, for tests on children, uncooperative patients, or patients needing assistance). For these reasons, in order to make possible a complete evaluation of the risks related to exposure to GMF in the magnet room, it is advisable to identify the clearance areas around the gantry of each tomograph. These areas, according to the approach proposed by Annex E of the EN 50499:2019-10 standard [13], are defined as follows.

Zone 0: Workplace in which exposure levels comply with the RLs for the general public (for example, paediatric caregivers; workers who are not professionally exposed and workers who are expected to carry specific risks) or all the equipment in the workplace is included in Table 1 of EN 50499:2019-10 [13]. This is a free access area for anyone.

Zone 1a: Exposures may be greater than the RLs for the general public but are compliant with Low ALs or sensory effect ELVs if relevant.

Zone 1b: Exposures are compliant with High ALs or health effect ELVs, but exceed any applicable sensory effect ELVs or Low ALs. Control measurements should be in place to ensure that any exceedance of the sensory effect ELVs is only temporary. The protective measures specified in Article 5, Paragraph 6 of the Directive 2013/35/EU [10] should be taken in case the low ALs for the electric field were exceeded.

Zone 2: Exposures may be greater than High ALs or health effect ELVs, and protective measures to reduce exposure or to restrict or limit access should be taken.

GMF signals emitted by MRI tomographs are characterised by complex waveforms with spectral content up to a few kilohertz. Therefore, the weighted peak method (which considers the different spectral contributions taking into account the respective phases and the relative regulatory limits) was used as a metric for the radiation protection interpretation of magnetic induction measurements. The method makes it possible to obtain a radiometric index [14,15] whose value, expressed as a percentage, indicates, depending on whether it is less than or greater than 100, respectively compliance with or violation of the regulatory limits taken as a reference for its calculation.

Furthermore, for two of the three tomographs analysed, we had the data from the respective manufacturers regarding the GMF emissions, so a comparison was made between the safety distances for workers and the population evaluated on the basis of the measurements made.

### 2.4. Measurement Instrumentation

Three different modes of acquisition and processing of GMF signals were used for the measurements, with two different instruments: a conventional commercial probe and a measurement chain specifically developed during previous research activities [2,3,4,5,6,7,8,9,10,11,12,13,14,15,16].

**Figure 2:**
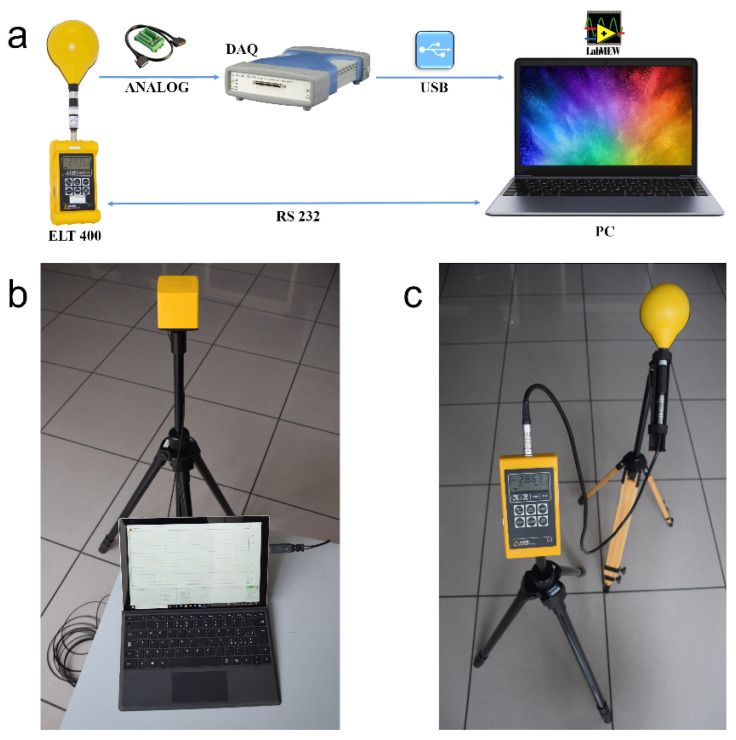
Instrumental chains for GFM measurement: (**a**) Narda ELT-400 with acquisition system; (**b**) Narda ELT-400 in a stand-alone version; (**c**) Narda-PMM EHP-50F.

The first method involves the use of a specific chain consisting of a Narda ELT-400 Exposure Level Tester (Narda Safety Test Solutions GmbH, 72793 Pfullingen, Germany) with a 100 cm^2^ triaxial sensor, an Agilent U2531A data acquisition device (Agilent Technologies Inc., Santa Clara, CA 95051, USA) connected to the analogue output of the probe, a portable personal computer connected via USB interface to the acquisition device and finally a software application created in Labview 2009 (National Instruments Corp., Austin, TX 78759, USA) for system management and the storage of the acquired data (Figure 2a).

This method allows the raw data acquired from the measurement chain to be acquired and processed offline, determining the weighted peak index relating to any other regulatory limit. For this purpose, specific processing software was implemented and made available on the WEBNIR platform. This software [17] makes it possible to elaborate the GMF measurements acquired with the presented instrumental chain and to determine the clearance from the source through the interpolation of the data measured along a straight line at progressively increasing distances [18].

The second method involves the use of only the tester previously inserted in the chain described, which, if used standalone, allows for the direct measurement of the magnetic induction and the weighted peak index relating to the RLs for the population envisaged by Recommendation 1999/519/EC [11] (Figure 2c).

The third method involves the use of a Narda-PMM EHP-50F Field Strength Analyzer (Narda Safety Test Solutions Srl, 17035 Cisano on Neva (SV), Italy). It consists of a triaxial probe for measuring electric and magnetic fields, capable of performing a spectral analysis via Fast Fourier Transform (FFT) in real-time, and to determine the weighted peak index in the time domain in relation to the main European and international regulations. The instrument is connected through a bidirectional fibre optic cable and an opto-electronic interface to a personal computer equipped with specific acquisition and control software (Figure 2b).

### 2.5. Limitations of These Methodologies

The main limitations of these methodologies are:

- The use of the measurement chain allows very accurate sampling for a post-analysis capable of giving a lot of information, such as the ability to calculate the weighted peak for the different levels of exposure. However, this chain is not marketed already assembled but requires experienced staff for the selection of components, assembly, as well as software development and management.
- The development of already-compact instruments able to directly provide the weighted peak index is spreading more and more and, despite their sampling capabilities are not as performing as the measurement chain described, they are quite reliable. However, these tools currently do not allow the saving of raw data for accurate post-analysis.

## 3. Results

The tables below show the values of the weighted peak indices expressed as a percentage, relating to compliance with ALs and RLs for magnetic induction according to the applicable European regulations. The modest differences in the percentage index detected with the various measurement methods are attributable to the reproducibility error in the positioning of the probes, their size, and the geometry of the sensors, without prejudice to their isotropy.

### 3.1. Tomograph Philips Panorama 1 T

The results relating to sequence A2 (Table 4 and Table 5 relating to 1 T tomograph), show that the RLs for the population and ALs for the workers are always respected even in the vicinity of the mouth of the magnet. Therefore, “Zone 0” according to EN 50499:2019-10 [13] for exposure to GMF is extended in this case to the entire magnet room.

### 3.2. Tomograph Philips Ingenia 1.5 T

**Figure 3:**
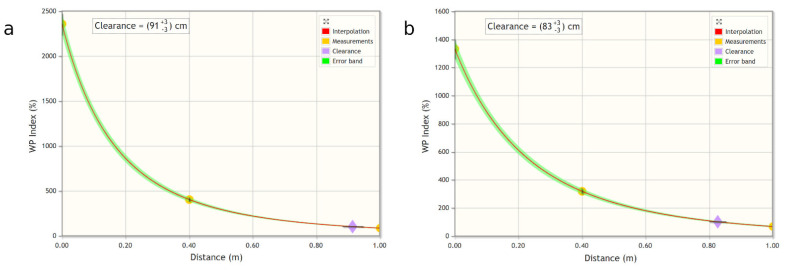
Interpolation of the B2 sequence at different distances and heights from the ground: (**a**) 100 cm from the ground (**b**) 160 cm from the ground.

Regarding the 1.5 T Ingenia tomograph, the ALs for workers have been respected, but there is an overrun of the RLs for the population. For this reason, it was decided to interpolate, with the appropriate WEBNIR tool, the results relating to the B2 sequence at the various distances (Table 6 and Table 7), and to calculate the free spaces for the general population at the two measurement heights (Figure 3).

The resulting values are 91 ± 3 cm at 100 cm from the ground and at 83 ± 3 cm at 160 cm from the ground. These distances identify the boundary between “Zone 0” and “Zone 1a” according to EN 50499:2019-10 [13].

### 3.3. Tomograph Siemens Magnetom Aera 1.5 T

Finally, the ALs for workers were also respected for the 1.5 T Aera Magnetom Tomograph, but there is an overrun of the RLs for the population. In this case, the interpolation of the results as a function of the distance related to the sequence C2 (Table 8), a clearance value was obtained for the individuals of the general population is obtained equal to 104 ± 3 cm. This distance identifies the boundary between “Zone 0” and “Zone 1a” according to EN 50499:2019-10 [13].

## 4. Discussion

The results of the measurements performed around the open magnet of the 1 T tomograph A) show that even in a point close to the mouth of the gantry, the RLs for the population and the ALs for workers regarding magnetic induction are always respected.

For the two 1.5 T cylindrical closed magnet devices (B) and (C), we highlight the need to keep a minimum distance of about 100 cm from the mouth of the gantry to ensure respect of the RLs envisaged for the population.

For staff, magnetic induction exposure levels were always compliant with the applicable ALs. There are no significant differences between the weighted peak indices referring to the High ALs and the Low ALs, demonstrating that the spectral content of the GMF signals is mostly above a frequency of 300 Hz, from which the two limits coincide.

Please note that “for magnetic fields, ‘low ALs’ means levels which relate to the sensory effects ELVs and ‘high ALs’ to the health effects ELVs”.

The assessment of the level of exposure to the electric field (not reported in this paper) does not highlight at any point of the measurement significant risk conditions for individuals of the population, least of all for professionally exposed staff.

The data provided by the manufacturers of the 1.5 T tomographs report the intensity trends of magnetic induction for diagnostic gradients, normalised to the centre of the gantry, according to the distance. The graphs show that the trends calculated following the measurements performed can be superimposed on those of the manufacturers, as well as the indicated safety distances. The comparison between the weighted peak index measurements performed with the ELT-400 tester in standalone mode, with ELT-400 connected to the external instrumental chain and with the EHP-50F analyzer, shows an acceptable correspondence between the different measurement systems.

Due to the limited availability of the MRI equipment, the measurements performed were not sufficient to reconstruct the distribution of the weighted peak index for the GMF across the area of the magnet room, as it would have been necessary to have a complete mapping or an exhaustive determination of the “clearance zone”. In terms of zoning according to the standard EN 50499:2019-10 [13] and taking into account the documentation provided by the manufacturers, even in the absence of a complete mapping, the method used made it possible to define, for the MRI equipment examined, the following semi-circular areas centred on the axis of the bed.

− Zone 0: over 100 cm away from the mouth of the magnet.
− Zone 1a: from the mouth up to 100 cm away from the magnet.

Thanks to the results obtained, it was possible to give useful indications for the protection of healthcare personnel and any accompanying persons of the patients that should remain in the magnet room during an examination: they could be made aware of the distance from the magnet at which to stop so the permitted level of exposure to GMF would not be exceeded.

On the other hand, the measurements performed confirmed the validity of the method used for the evaluation of the weighted peak index for exposure to GMF, which can therefore be proposed as a general approach to be taken in one of the presented configurations, to perform a spatially more detailed evaluation or to explore in-depth the evaluation for specific sequences of GMF. Finally, the measurement points chosen inside the magnet room (0 cm, 40 cm, OP, and REAR), are those where a healthcare professional is usually stationed to assist the patient during a diagnostic examination.

It must be borne in mind that if the future foresees a wide diffusion of 3 T MRI and for more detailed diagnostics the use of 7 T tomographs, currently, for economic reasons, 1 and 1.5 T magnetic resonances are widely used for daily assessments. Furthermore, as already mentioned, these devices are used 24 h a day, and access for metrological surveys must be scheduled and is limited in time; for this reason, these types of studies are difficult to carry out and relatively rare.

Obviously, the future scenario of this study will also be the metrological evaluation of the GMF emitted by 3 T and 7 T magnetic resonances.

## 5. Conclusions

The method adopted for the measurements proved to be adequate to the needs of the evaluations to be carried out. With this method, one of the different types of instruments used and the WEBNIR platform, freely accessible via the Web, it should be possible to carry out in a simple way a sufficiently accurate assessment of the level of exposure to GMF within the magnet room of any MR equipment, even with a static magnetic induction value higher than 1.5 T.

The assessments performed provided clear indications in relation to the level of exposure to GMF of medical personnel (doctors, radiology technicians, anesthetists, and nurses) assigned to MRI tomographs up to 1.5 T. A reassuring scenario emerges under normal operating conditions, even when it is essential to remain inside the magnet room during acquisitions. A modest lateral movement of a few tens of centimetres from the mouth of the gantry is sufficient to ensure compliance with the occupational limits for magnetic induction, even during the fastest diagnostic sequences.

Any subjects of the population (patient carers) or non-professionally exposed workers who must remain inside the magnet room during an examination can be provided with adequate recommendations on maintaining a distance of at least one meter from the mouth of the gantry to prevent any reversible sensory effects of neuro-muscular stimulation. Should a closer distance be maintained in order to assist the patient, informed consent regarding the specific exposure to GMF for possible sensory effects must be acquired.

The safety limits recommended by ICNIRP guidelines and specified by EU standards incorporate a precautionary margin with respect to the thresholds of the effects, which also depend on the individual characteristics of the exposed subject. It is not possible to make a forecast other than conservatively by keeping the exposure below the limits.

## Figures and Tables

**Table 1:** European Directive 2013/35/EU—Action Levels for exposure to magnetic fields from 1 Hz to 10 MHz.

Frequency range	Magnetic Flux Density Low AL_S_ (μT) (RMS)	Magnetic Flux Density High AL_S_ (μT) (RMS)	Magnetic Flux Density AL_S_ for Exposure of Limbs to a Localised Magnetic Field (μT) (RMS)
1 ≤ *f* < 8 Hz	2.0 × 10^5^/*f*^2^	3.0 × 10^5^/*f*	9.0 × 10^5^/*f*
8 ≤ *f* < 25 Hz	2.5 × 10^4^/*f*	3.0 × 10^5^/*f*	9.0 × 10^5^/*f*
25 ≤ *f* < 300 Hz	1.0 × 10^3^	3.0 × 10^5^/*f*	9.0 × 10^5^/*f*
300 Hz ≤ *f* < 3 kHz	3.0 × 10^5^/*f*	3.0 × 10^5^/*f*	9.0 × 10^5^/*f*
3 kHz ≤ *f* ≤ 10 MHz	1.0 × 10^2^	1.0 × 10^2^	3.0 × 10^2^

**Table 2:** European Recommendation 1999/519/EC—Reference levels for electric, magnetic and electromagnetic fields (0 Hz to 300 GHz, unperturbed RMS values).

Frequency Range	E-Field Strength (V/m)	H-Field Strength(A/m)	B-Field(μT)	Equivalent Plane WavePower Density S_eq_ (W/m^2^)
0–1 Hz	—	3.2 × 10^4^	4 × 10^4^	—
1–8 Hz	10,000	3.2 × 10^4^/*f* ^2^	4 × 10^4^/*f* ^2^	—
8–25 Hz	10,000	4000/*f*	5000/*f*	—
0.025–0.8 kHz	250/*f*	4/*f*	5/*f*	—
0.8–3 kHz	250/*f*	5	6.25	—
3–150 kHz	87	5	6.25	—
0.15–1 MHz	87	0.73/*f*	0.92/*f*	—
1–10 MHz	87/*f*^1/2^	0.73/*f*	0.92/*f*	—
10–400 MHz	28	0.073	0.092	2
400–2000 MHz	1.375 *f* ^1/2^	0.0037 *f* ^1/2^	0.0046 *f* ^1/2^	*f*/200
2–300 GHz	61	0.16	0.20	10

**Table 3:** Tomographs and GMF diagnostic sequences analysed; the “Code” column shows the reference with which the sequences are indicated in the result tables.

Tomograph	Gradient Sequence	
	Code	Description
(A) Philips Panorama 1 T	A1	SPIN-ECHO, Repetition Time 729 ms, Slice Gap 0.35 mm.
	A2	STIR, Repetition time 2500 ms, Inversion Time 120 ms, Echo time 70 ms.
(B) Philips Ingenia 1.5 T	B1	SPAIR Lumbar Spine 200 mm, Echo Time 80 ms, Repetition Time 4179 ms, Suppression of fat.
	B2	STIR Long TE Lumbar Spine (Sagittal-Lumbar), Echo Time 60 ms, Repetition Time 2500–4000 ms, Inversion Time 160 ms.
(C) Siemens Magnetom Aera 1.5 T	C1	T1 Coronal TIRM P2-320 Bilateral, Repetition Time 5110 ms, Echo time 52 ms, Slice Thickness 4.5 mm.
	C2	TURBO SPIN ECHO FatSat 256 bilateral, Repetition Time 481 ms, Echo Time 7.3 ms, Slice Thickness 4.5 mm.

**Table 4:** Measurement results with the ELT-400 probe (Display values—Mode Exposure STD ICNIRP 1998 General Public—Range Low—Low Cut 30 Hz—Detector STND) and EHP-50F (Mode WP, magnetic field, full scale 100 µT).

Sequence	Position		Probe ELT-400	Probe EHP-50F		
	Distance (cm)	Height (cm)	RLs 1999/519/EC	RLs 1999/519/EC	Low ALs 2013/35/EU	High ALs 2013/35/EU
A1	0	100	46.6%			
A2	0		76.0%	70.0%	2.0%	1.5%
	50		7.0%	9.0%	0.3%	0.3%
	100		2.4%	2.4%	0.1%	0.1%
	150		1.7%			

**Table 5:** Measurement results with ELT-400 probe (postprocessing of data acquired from the analogue outputs—Mode Field Strength 320 μT—Range Low—Low Cut 30 Hz).

Sequence	Distance (cm)	Height (cm)	RLs 1999/519/EC	Low ALs 2013/35/EU	High ALs 2013/35/EU
A2	0	100	69.5%	3.36%	3.56%

**Table 6:** Measurement results with the EHP-50F probe (WP mode, magnetic field, full scale of 100 µT).

Sequence	Distance (cm)	Height (cm)	RLs 1999/519/EC	Low ALs 2013/35/EU	High ALs 2013/35/EU
B1	0	100	1389%	45.0%	45.6%
	OP		22.5%	0.70%	0.60%
B2	0	100	2356%	76.0%	72.5%
	40		401%	13.0%	12.0%
	100		83%	2.50%	2.10%
	OP		51%	1.57%	1.25%
	0	160	1332%	40.1%	40.8%
	40		317%	9.70%	9.17%
	100		66%	2.00%	1.65%

**Table 7:** Measurements results with the ELT-400 probe (Postprocessing of the data acquired from the analogue outputs—Mode Field Strength 320 μT—Range Low—Low Cut 30 Hz).

Sequence	Distance (cm)	Height (cm)	RLs 1999/519/EC	Low ALs 2013/35/EU	High ALs 2013/35/EU
B1	0	100	1423%	52.4%	53.2%

**Table 8:** Measurement results with the EHP-50F probe (WP mode, magnetic field, full scale 100 µT).

Sequence	Distance (cm)	Height (cm)	RLs 1999/519/EC	Low ALs 2013/35/EU	High ALs 2013/35/EU
C1	0	100	2032%	78.9%	86.5%
C2	0	100	2642%	88.0%	92.0%
	50		338%	11.4%	10.7%
	100		100%	3.36%	2.82%
	150		44.4%	1.51%	1.21%
	REAR		916%	31.0%	29.5%

## Data Availability

The datasets analyzed during the study are available from the first author or the corresponding author upon reasonable request.
